# Intermediate LET-like effect in distal part of proton Bragg peak revealed by track-ends imaging during super-Fricke radiolysis

**DOI:** 10.1038/s41598-023-42639-4

**Published:** 2023-09-19

**Authors:** J. Audouin, P. Hofverberg, Y. Ngono-Ravache, L. Desorgher, G. Baldacchino

**Affiliations:** 1 Université Paris-Saclay, CEA, CNRS, LIDYL, 91191 Gif-sur-Yvette, France; 2 Centre Antoine Lacassagne, 06100 Nice, France; 3 CIMAP, CEA-CNRS-ENSICAEN-UNICAEN, Normandy University, Cedex 04, 14050 Caen, France; 4 Institute of Radiation Physics (IRA), Lausanne University Hospital and University of Lausanne, CH-1007 Lausanne, Switzerland

**Keywords:** Radiotherapy, Energy transfer, Reaction kinetics and dynamics, Spectrophotometry, Imaging and sensing

## Abstract

Upstream of the efficiency of proton or carbon ion beams in cancer therapy, and to optimize hadrontherapy results, we analysed the chemistry of Fricke solutions in track-end of 64-MeV protons and 1.14-GeV carbon ions. An original optical setup is designed to determine the primary track-segment yields along the last millimetres of the ion track with a sub-millimetre resolution. The Fe^3+^-yield falls in the Bragg peak to (4.9 ± 0.4) × 10^–7^ mol/J and 1.9 × 10^–7^ mol/J, under protons and carbon ions respectively. Beyond the Bragg peak, a yield recovery is observed over 1 mm for proton beams. It is attributed to the intermediate-LET of protons in this region where their energy decreases and energy distribution becomes broader, in relation with the longitudinal straggling of the beam. Consequently to this LET decrease in the distal part of the Bragg peak, Fe^3+^-yield increases. For the first time, this signature is highlighted at the chemical level under proton irradiation. Nevertheless, this phenomenon is not identified for carbon ion beams since their straggling is lower. It would need a greater spatial resolution to be observed.

## Introduction

Hadrontherapy is known for its great relative biological effectiveness (RBE) and oxygen enhanced ratio (OER) for the treatment of deep localized tumours. These are generally hypoxic tumours for which a radio-resistance has been observed very early and described as an “oxygen effect” under X-rays^1–3^. This RBE stems from the increase of the ion energy deposition efficiency with decreasing ion energy until a maximum value is reached just before the ion completely stop. This maximum is called the Bragg Peak (BP)^4–6^. The ion track end is typically characterized by an elevated electronic Linear Energy Transfer (LET). The LET represents the variation of the particle energy –d*E*, through electronic interactions, over an infinitesimal step d*x*^7^. The Bethe-Bloch theory helps for determining the ranges and LET (=− d*E*/d*x*) as a function of both particle and matter characteristics^8^. LET at the BP is in the order of a few 10 to a few 1000 keV/µm for high-energy protons or carbons through liquid water. The SRIM application provides the necessary calculations of the LET along the propagation range^9^. High LET particles ionize and excite water molecules along their trajectory in a dense and structured track^10,11^. Following this early water radiolysis physical stage (ionizations and excitations of H_2_O), intense physical chemistry processes occur^7,12–14^. Those are comparable to a huge dose rate effect but localized in the ionization track structure^10,15,16^. As a chemical consequence, molecular oxygen can be formed in these dense tracks^17^. Several reaction pathways can lead to the formation of molecular oxygen in water radiolysis when high-LET projectiles are used^17^. This was depicted in several studies and very few concern the effects in isolated BP in liquid water^18,19^. The oxygen provided in this way can participate to locally reduce the hypoxia for instance in radio-resistant hypoxic tumours.

Currently, chemical effects in BP can address many topics in fields of primary concern: radiation protection and dosimetry^20^, space radiation protection^6,21^, alpha rays in nuclear industry^22^, alpha and Boron neutron capture therapy (BNCT)^23,24^. To take advantage or to protect from damage occurring in BP, understanding the radiation chemistry in this region with a high spatial resolution is necessary. This requires observing fast chemical processes within a small volume, typically in the mm^3^ range. Unfortunately, the literature does not dive in the details of the chemistry within the BP despite suspected peculiar processes encountered in this “hot region” of dose delivery in relation with the LET and RBE values adopted in protontherapy^25^. Experimental and theoretical approaches remain crucial challenge in this domain for which we pretend to give a new insight.

The parameter accounting for the track effect and the density of ionization in the BP is the primary yield (G-value) of radiolytic chemical species^16^, which can be radicals, ions (resulting from a reduction or an oxidation), and molecules formed by radical recombination. Many experimental studies in the literature report the effect of LET on primary radiolytic yields of hydroxyl radical (^⋅^OH), hydrated electron (e^─^_aq_), ferric ion (Fe^3+^) and hydrogen peroxide or molecular hydrogen, to cite only the radiolytic species of the highest interest in the wide water-radiolysis literature^7^. As the LET value of an energetic ion in water evolves along the ion track, considering the track as a whole will lead to an average value of the yield. On the opposite, the yield will be a track-segment yield or a differential yield^7,18,19,26^ in regions where the LET does not vary too much. These are generally determined by changing the ion beam energy and by analysing the concentration of radiolytic species of interest^18,19^. This also requires the mathematical reconstruction of G-values as a function of the propagation coordinates from the G-values determined as a function of the beam energy^27^.

This method was exploited by Maeyama et al*.* in 2011 to measure the hydroxyl radical yields in BP by using ions of very high energies, above GeV^28^. These authors used 1 cm of water solution to analyse the production of ^⋅^OH and they lowered the initial beam energy by interposing poly-methyl-methacrylate (PMMA) foils of increasing thicknesses, with a precision higher than 0.6 mm. Their experimental results have been compared to Monte Carlo simulations provided by the PHITS code and revealed the effects of the fragmentations, which are nuclear processes occurring above 1 GeV. This process provides particles of greater LET. Nevertheless, details in the formation of radiolytic species as a function of the dose distribution along the ion path, in particular at the BP, are required with a high spatial precision to optimize the local chemistry occurring in hadrontherapy using proton or carbon ions for the tumour treatment.

More recently, calculations using the GEANT4-DNA Monte Carlo code have reproduced the yields resulting from high ionization densities in the BP region for various energetic ion beams in water^19^. The authors challenged the difficulty resulting from the high ionization density and the multiplicity of the events, which force time-consuming simulations. They proposed a strategy that succeeded in explaining experimental results in terms of differential G-values but still without a sub-millimetre resolution^18,29^.

Much in relation with hadrontherapy performance, Horendeck et al. have observed high LET-like track structures in micrometre size cells in the post-BP region of a 70-MeV proton beam, the proton beam stopping within a 5-cm large vessel. The track structure, presented as localized damage in γ-H2AX cells, looks like short tracks resulting from spurs overlapping. This track structure actually looks like a track of high-LET particle producing similar damage structures. These observed damages might explain sides effects reported in protontherapy^30,31^. Their observations are in line with those observed on RBE values recently obtained for proton therapy beams^32–35^.

Therefore, going further in the description of the BP through the specific chemical processes under very high density of ionization is relevant to depict the upstream reactivity of the biological step. This implies to identify the reactive species formed through the radiolysis process at earliest times after the passage of the ion beam, through the use of pulsed beam^7,36^. This detection has also to be performed along the track, in the small region of the BP. Another method would consist in taking into account the primary yield of species of interest. The primary yield represents the limit to dose 0 Gy of the ratio between the concentration *C* of the species over the dose *D*, i.e. $\underset{D\to 0}{\mathrm{lim}}\left(C/D\right)$. This can be obtained under a continuous stable beam or several repeated pulses in a homogenised solution^16^. An example of this yield determination was given recently^17^. The challenge is to carry out a similar detection to obtain the yield determination at the same time along a few millimetres along the propagation axis of the ion track-end in liquid water.

Thus, we propose a novel experimental setup to analyse inline radical formation as a function of the dose along the track of an energetic ion, until it stops, in a liquid water sample. We will focus in this article on the sensitive Fricke dosimeter system, which gives a whole behaviour of water radiolysis by combining the primary yields of ^⋅^OH, e^─^_aq_, H_2_O_2_, ^⋅^H, and HO_2_^⋅^. This proof of concept was preferred to the study of each individual radical or molecular species such as H_2_O_2_. The reason is that they are expected to be very fleeting as these species are transient due to their high reactivity. Besides, they have very different absorption coefficients: low for ^⋅^OH, in UV domain and high for e^─^_aq_ in the NIR domain^37^. In particular, the ferric ions yield for the Fricke dosimeter system is known to be sensitive to the LET^13,38,39^.

Moreover, by associating a multi-channel detection to a collinear light transmission technique in an aqueous solution along the proton-beam propagation-axis, yields can be obtained on ultra-short track-segment with a submillimetre resolution without mathematical reconstruction. This accuracy is commonly adopted for the localization of the BP^40^. In this article, we present and discuss the results obtained with this setup for 62-MeV and 25-MeV proton beams and carbon beams of less than 1 GeV of energy, propagating and stopping within 1 cm of liquid water.

## Results

### Super-Fricke solution absorption under protons and carbon ions, along the propagation axis

**Figure 1:**
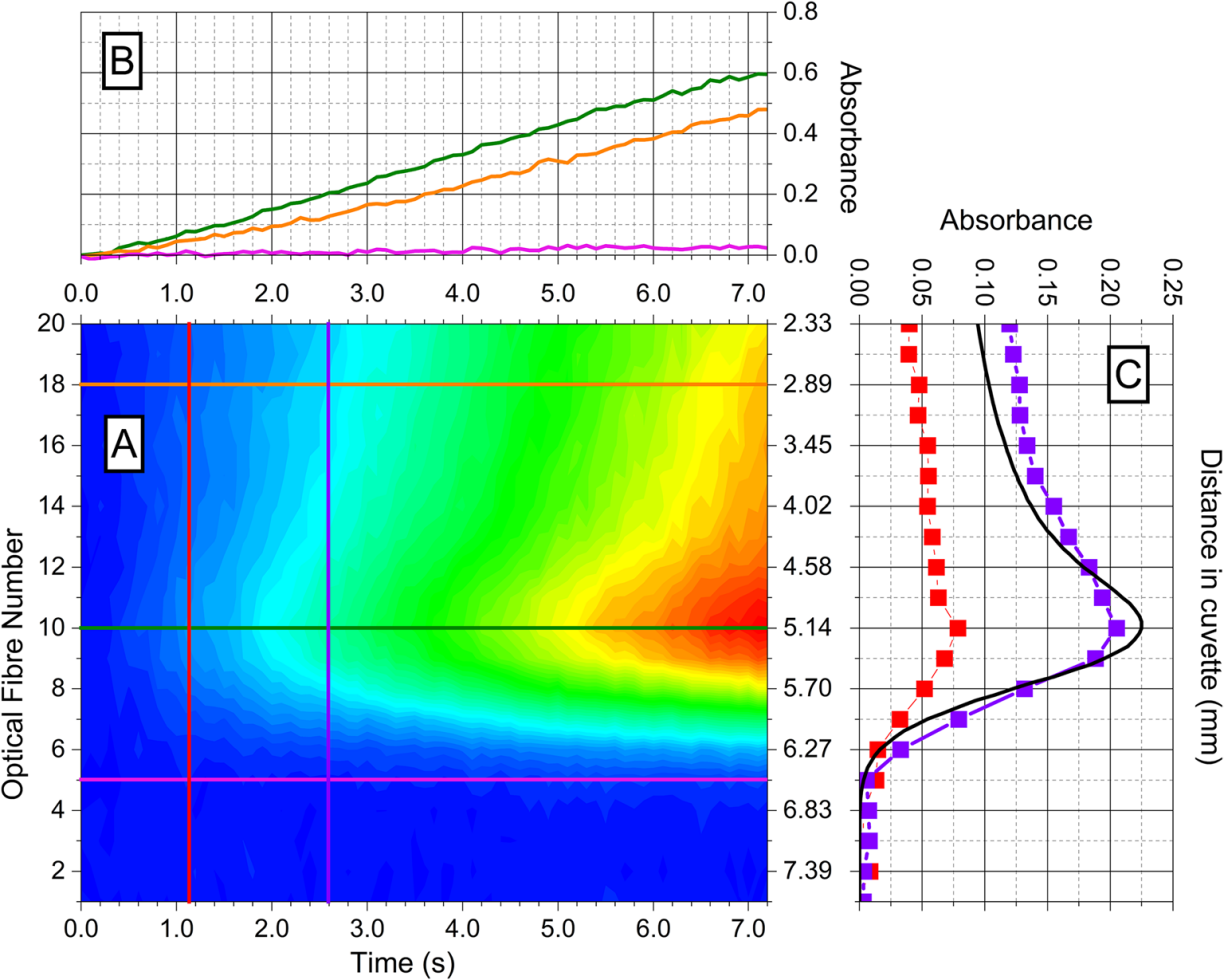
Super-Fricke absorption exposed to a 25-MeV proton beam of 0.5 nA over time. (**A**) Absorption during time along the propagation axis. Dose rate at BP is evaluated at 70 Gy/s. (**B**) Absorbance over the 7 first seconds of the irradiation respectively for (pink line) fibre 5 (green fline) fibre 10 and (orange line) fibre 18. (**C**) Absorption along the proton propagation axis for the 20 fibres at 2 selected times: (red filled square) at 1.1 s and (violet filled square) at 2.6 s. (Black line) is the normalized ELF curve of a 25-MeV proton beam in a Super-Fricke solution calculated with TRIM.

Figure 1 presents the selected image sequence in the first 7 s of a super-Fricke solution irradiated with a 25-MeV proton beam at an intensity of 0.5 nA. The 3D-picture (A) shows the absorbance as a function of time and along the proton propagation axis. The fibre at position 20 corresponds to the beam entrance with 25 MeV of energy. (C) shows that fibre 1 signal is not affected by any dose deposition since the proton beam stops before; as the BP is located around position 10. (B) shows that during the first moment of irradiation, the absorption growth is linear with the dose on fibres 10 and 18. It also highlights the onset of a saturation-like behaviour around 7 s on fibre 10. This behaviour is expected because the dose rate in the BP for a 0.5 nA proton beam is estimated at 70 Gy/s. Thus, the dose received in this region exceeds 500 Gy after 7 s of irradiation, which is the highest limit of dose for the Fricke dosimeter with low LET particles, according to literature, even if this limit is pushed back for super-Fricke (vide infra)^41^. The diffusion process also starts to become significant and participates to the expansion inside the whole 3-mL solution of the Fe^3+^ species formed during the first 7-s beam exposure. We can observe this diffusion phenomenon on fibre 5 as a slow absorption increase. The plot presenting the energy loss function (ELF) in (C), calculated with TRIM, shows that there is no dose deposited before fibre 5, hence the absorption is mainly due to the diffusion of earlier formed Fe^3+^. The diffusion of species out of the 8 mm diameter cylinder is a process that occurs as soon as species are formed. Given that Fe^3+^ has a diffusion coefficient of 6.07 × 10^–10^ m^2^/s at 25 °C^42^, the order of magnitude of the increase during 1 s of the initial section of 8 mm would be 28 µm. This would represent a variation of more than 10% of the Fe^3+^ concentration, after 28 s. The linearity of the absorption’s growth during the first seconds of the irradiation is observed along the entire track but with different slopes. The steepest slope is positioned at the BP (fibre 10), and the slope tends to decrease when the distance from the BP increases (cf. fibre 18). (C) demonstrates that during the first seconds of irradiation, the shape of the absorption curve along the track keeps its form through time with a maximum on fibre 10. This maximum of absorption coincides with the maximum of the ELF curve (i.e. with the BP). We suppose that the precision of the BP localisation is about a few hundreds of µm, which is from one hand below the resolution of an optical fibre and on the other hand, corresponds to the accuracy between two irradiation sequences (Cf SI-5 “Estimation of error bars”). Overall, we estimate a BP-position with an accuracy of less than 1 mm.

It is not relevant to comment the quantitative values of absorption themselves and the subsequent concentrations of the formed species (Fe^3+^) because they are strongly correlated to the dose rate (i.e. the beam intensity) in the sample during time.

**Figure 2:**
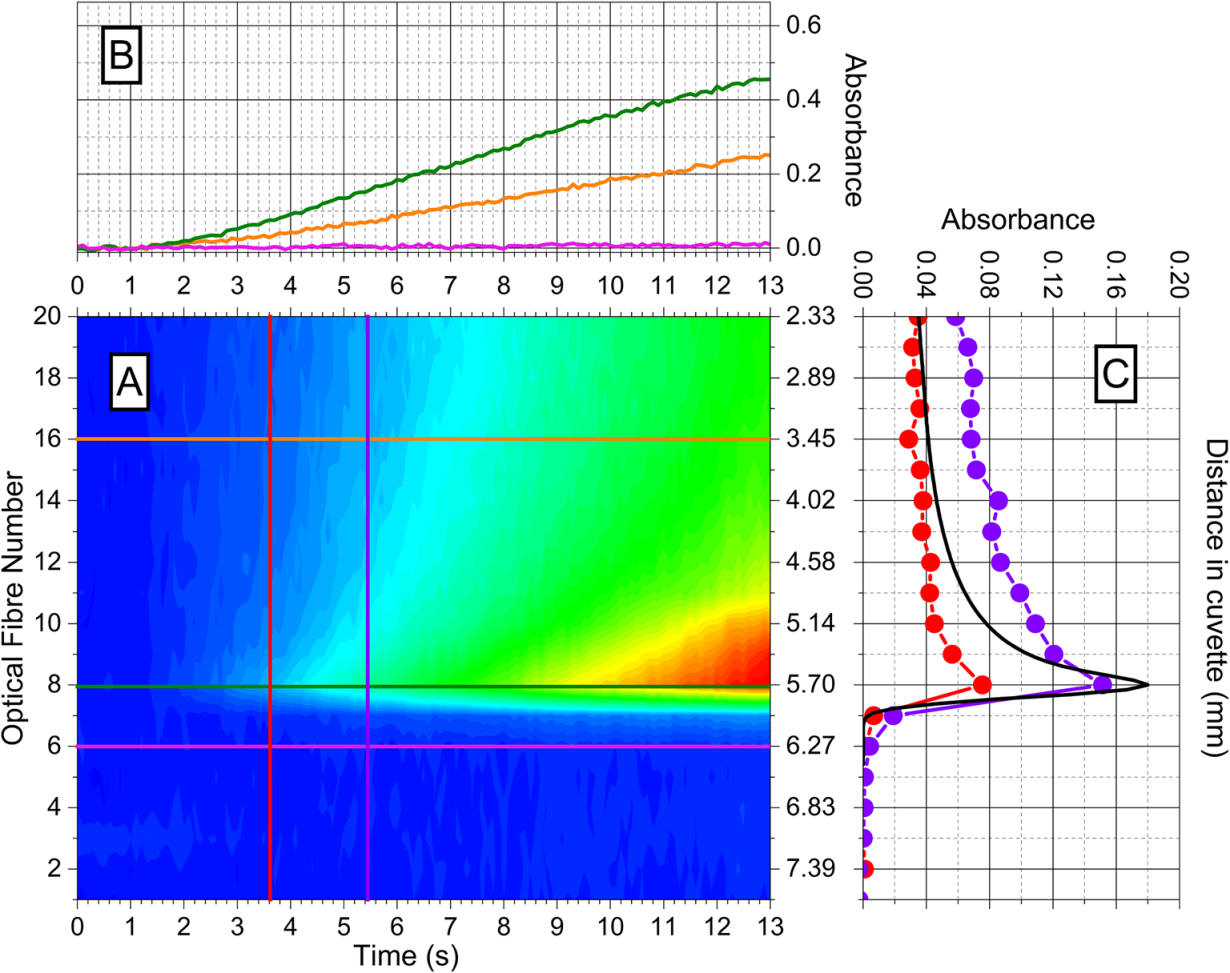
Super-Fricke absorption exposed to a 550 MeV-carbon ion beam of 0.1 nA over time. (**A**) Absorption during time along the propagation axis of carbon beam in a super-Fricke solution. The dose rate at BP is evaluated at 88 Gy/s. (**B**) (pink line) fibre 6, (green line) fibre 10, (orange line) fibre 18. (**C**) (Red filled circle) at 1.1 s, (violet filled circle) at 2.6 s. (Black line) is the normalized 550 MeV-carbon ion ELF curve in a super-Fricke solution calculated with TRIM.

Figure 2 presents the selected image sequence in the first 13 s of a super-Fricke solution irradiated with 550-MeV carbon ions at a beam intensity of 0.1 nA. (A) displays the absorbance as a function of the irradiation time and along the carbon propagation axis. (C) exhibits a BP localized around fibre 8 at the maximum of absorption. There is no absorption on fibre 6 since the carbon beam is stopped, at the farthest, on fibre 7. (B) shows a linear growth of the absorption as a function of the time similar to the one observed for protons, with an onset of a saturation-like behaviour after 12–13 s of irradiation. Similar to what is observed with proton beam, the coincidence of the maximum of the ELF curve with the maximum of absorption in the track is observed between the increase of absorption along the track and the ELF curve in the early times of irradiation. It also reveals that the BP is much thinner with the carbon beam than for the proton beam. In Fig. 1, we could observe absorption signal from 4 fibres after the BP while in Fig. 2 we observe absorption from only 1 fibre after the BP. The BP is also more intense for carbon ions. If we compare the value of absorption at the beginning of the track (fibre 20) with the maximum value at the BP, we obtain a ratio higher than 2 for carbon ions (2.17 and 2.59 respectively for red filled circle and violet filled circle) and lower than 2 for protons (1.98 et 1.71 respectively for red filled square and violet filled square).

### Super-Fricke track-segment yields in the track-end of protons and carbon ions

**Figure 3:**
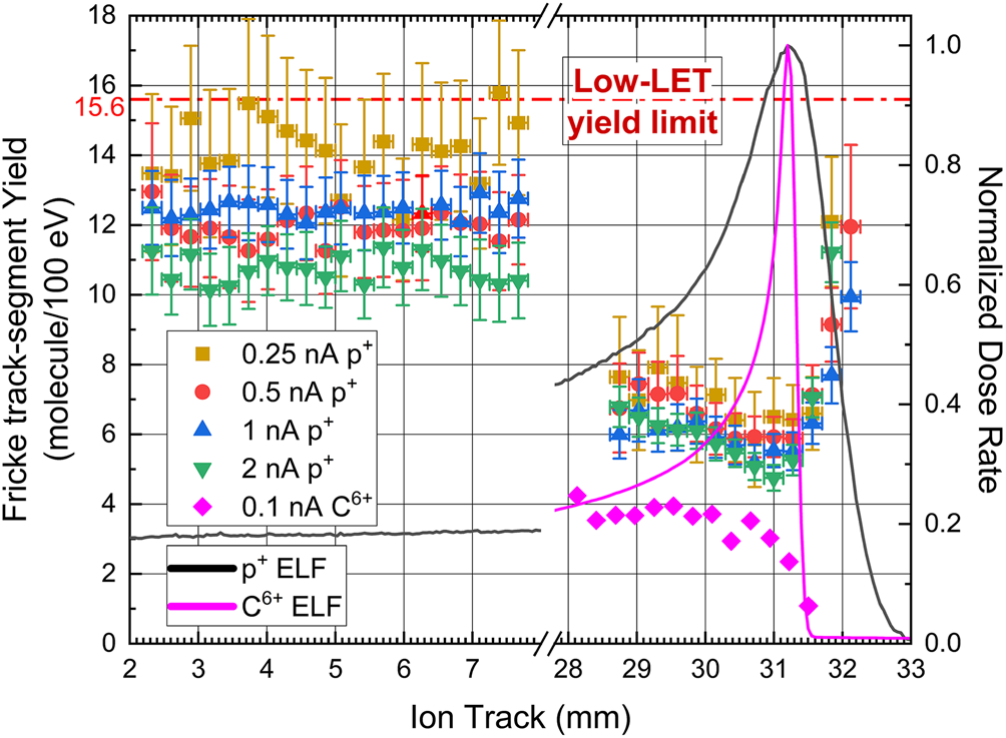
Track-segment yields of Fe^3+^ in Super Fricke solution for 62-MeV protons with intensity of 0.25 (yellow filled square); 0.5 (red filled circle); 1 (blue filled triangle) and 2 nA (green filled inverted triangle), and 550-MeV carbon ions of 0.1 nA (pink filled diamond). (Black line) ELF for a proton beam calculated with GEANT4. (Pink line) ELF for a carbon ion beam calculated with GEANT4. For clear presentation, we have arbitrary shifted the carbon plots in a manner to make coincide the Bragg peaks of the proton and the carbon.

The primary track-segment yields, G(Fe^3+^), presented in Fig. 3 were computed according to Eq. (4), from the absorption data and the ELF calculated with the GEANT4 Monte Carlo code. Even if TRIM, GEANT4 and FLUKA give similar values and shape of the ELF (Cf. SI-1), especially in BP, we prefer to consider the beam geometry and to make available an estimation of the accuracy of the yields (Cf. SI-5). TRIM neither considers the geometry of the beam (like the beam section), nor the distribution profile at the entrance to the sample cuvette, nor the energy spectrum of the beam. TRIM ELF presented in Figs. 1 and 2 only served as illustrations. In addition, as we were able to perform 2 irradiations with the same parameters for each beam intensity, we calculated the yields as a mean of the 2 experimental values. We also assume to have some fluctuating parameters that affect the experiment repeatability. In particular, the initial mean energy of the proton beam, the cuvette window thickness and the sample thickness are parameters that can change significantly enough to modify the position of the BP from one acquisition to another and from one sample cuvette to another. Practically, that means that the positions of the BP and of the mean maximum absorption in the track slightly change from one beam intensity to another between fibre 10 and 12 with a position centred on fibre 11.

In this context, it is rather difficult to position precisely the ELF, which evolves very quickly near the BP. Thus, if we use the same ELF curve (with the BP localized on fibre 11) to calculate all G(Fe^3+^), very different shapes and values for G(Fe^3+^) are obtained from one intensity to the other as the experimental BP does not coincide with the calculated BP for each intensity. We use these variations to estimate an error bar for the fibre position (Cf SI-5), which could be translated into an energy accuracy, or a BP position accuracy.

The carbon yields presented in Fig. 3 are preliminary results that were obtained similarly to the proton yields. Each point is a mean value of 2 experimental values. They were computed using ELF given by Geant4 simulation. The maximum coincides with the fibre 5, which is a region where no absorption was observed by the experimental setup (Fig. 2). Therefore, we chose to adjust the maximum of the ELF with fibre 8 where the variation of absorption is maximal, which likely corresponds to a region where the dose rate is maximum. We did that to quickly observe the consequences of a much thinner and more intense Bragg peak on the yields and to identify further perspectives of study. No error bars have been estimated due to the earliness of the results.

Figure 3 shows that the shape of the G(Fe^3+^) curves obtained for all the intensities are very similar, especially in the BP area. On the left part of the break, i.e. the low LET region (also low ELF region, or high energy proton region), we observe high values of G(Fe^3+^) varying from 10.6 to 16.4 molecule/100 eV (respectively 11.0 × 10^–7^ mol/J to 17.0 × 10^–7^ mol/J in SI). The average values in molecule/100 eV of 14.9, 12.5, 13.1 and 10.6 correspond respectively to intensities of 0.25 nA, 0.5 nA, 1 nA and 2 nA. A decrease tendency is observed on this part of the track but without a clear beam intensity effect. The discrepancy in the yield values should rather be correlated to the initial absorption signal-to-noise ratio (and consequently to the initial slope) which is low when using low beam intensity. On the right part of the break (i.e. for low proton energy, including the energies in the BP), the G(Fe^3+^) values are much lower and are very close for all the intensities. This latter behaviour corresponds to the increase of the signal-to-noise ratio in relation with a greater absorption, which eases the initial slope determination compared to what observed for the lowest LET range. At the 28–29 mm position from the track beginning, the values are between 6.0 and 7.6 molecule/100 eV (respectively 6.2 × 10^–7^ mol/J to 7.9 × 10^–7^ mol/J in SI), which almost represent half of the values obtained on the left part of the plot. Further in the track, G(Fe^3+^) values continue to decrease (as the ELF curve increases) until they reach minimum values between 4.75 and 6.51 molecule/100 eV (respectively 4.9 × 10^–7^ mol/J to 6.7 × 10^–7^ mol/J in SI), respectively obtained for beam intensities of 0.25 and 2 nA. After the minimum, a marked rise of the yields takes place in the post-BP region. This is mostly visible for the 1 and 2 nA plots because of their improved signal-to-noise ratio. In this domain, some data are removed from the plots related to irradiations at 0.25 and 0.5 nA (yellow and red points respectively) because they are out of range. G(Fe^3+^) values then reach 10–13 molecule/100 eV (10^–6^–1.3 × 10^–6^ mol/J in SI).

The carbon yields on Fig. 3 shows a similar trend: the left part of the track where LET are still elevated but not at the maximum like in BP, reveals low yield values with the first value being at 4.2 molecule/100 eV (4.3 × 10^–7^ mol/J). They are similar to what the literature proposes^38^. The yield decreases along the track. The value corresponding to the maximum of the ELF curve is at 2.4 molecule/100 eV (2.5 × 10^–7^ mol/J). Moreover, the last point in the post-BP region is even lower, at 1.1 molecule/100 eV (1.1 × 10^–7^ mol/J). The yields for carbon beams in the BP region are about half the yields obtained for proton beams. Since a ratio of more than 6 is noticed between the dose rate value in BP of carbon compared to the dose rate in proton BP for the same beam intensity, a much higher density of ionization and excitation observed with carbon beam can explain this factor of 2. However, we observe no increase of the yields in the post-BP region for the carbon beams. As the BP area is narrower, the current spatial resolution of our optical setup is probably too low to be able to observe a potential increase in yields in carbon tracks in this region.

## Discussion

Figure 3 summarizes the behaviour of the super-Fricke solution in the track end of protons and carbon ions. As it is well discussed in the literature, the decrease of the species yield in the BP is a consequence of the elevated ionization density provided by the extreme LET values in this part of the track^38,39^. These newly reported results present segment-track yield values for high energy proton (left part of the Fig. 3 corresponding to 62-MeV protons) below the low-LET yield limit of 15.6 molecules/100 eV (16.1 × 10^–7^ mol/J) mentioned in the literature. The minimum track-segment yields of 5 molecules/100 eV (5.2 × 10^–7^ mol/J) (with an estimated accuracy of 0.2 molecules/100 eV- i.e. 0.2 × 10^–7^ mol/J) and 2 molecules/100 eV (2.1 × 10^–7^ mol/J) at the proton BP and the carbon BP, respectively, provide a new extension of the experimental results provided by Pimblott and LaVerne^39^. They conclude on these very low values by using Monte Carlo simulation of the track structure and the early chemical processes. In particular, they highlighted the scavenging reactions of H_2_O_2_ and HO_2_^⋅^ formed in intra-track bimolecular recombination of ^⋅^OH radicals on one hand and recombination of O_2_ and e^─^_aq_ on the other hand.

In addition, the description of the ion track-end obtained with a respectable signal-to-noise ratio, depending essentially on the beam intensity, and with a sub-millimetre resolution, reveals some details never mentioned in the literature.

**Figure 4:**
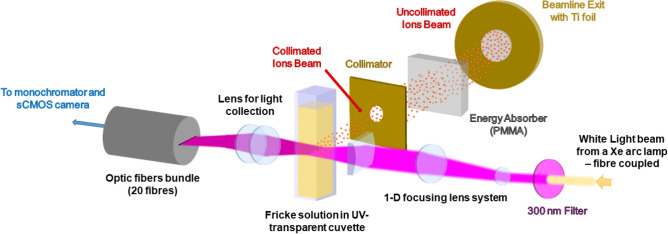
Schematic of the optic setup used during experiments with ionizing beams. Same setup is used for proton and carbon ion beams.

The posterior part of the BP, where the ions are supposed to stop, seems to be characterized by an increase in the yield values in the nearest millimetre after the proton BP (Fig. 3). This is not visible for the carbon ions for which G(Fe^3+^) are also mentioned in Fig. 3. Since the BP of carbon ions is sharper than that of protons, we assume that the spatial resolution is not sufficient to describe this non-negligible but decreasing dose rate range to zero. We refute that the post-BP behaviour is the result of the fragmentation process which has often been evoked and which is, however, to be taken into account at energies higher than GeV^28^. Otherwise it should be more remarkable for carbon ions. Thus, this behaviour is not the result of the LET value itself but only the result of the decrease of the overall dose rate after the BP. The origin of this phenomenon could be the longitudinal extension of the BP dose rate, called longitudinal straggling. This straggling is significant for the protons crossing a stack of several materials (Cf SI-1)^43^. This phenomenon does not affect too much the carbon ion BP. The maximum dose rates, corresponding to the BP, for a proton beam of 1 nA and a carbon beam of 0.1 nA are 140 Gy/s and 88 Gy/s respectively. Within 4 s for proton and 6 s for carbon, the doses reach values greater than 500 Gy, which in literature, is the estimated limit dose available for using Fricke dosimeter considering low LET particles (γ-rays or energetic electrons)^44^. Extension to 10^3^ Gy and 10^7^ Gy/s is available for super-Fricke solution^41^. Thus, despite the elevated dose rates in the BP, the determination of the yield in the first 4 s of the irradiation remains in the linearity domain of the super-Fricke response. Therefore, a lower dose rate in post-BP cannot completely explain the observed rise of the yield. This is always true for the reduced dose rates in the distal part of BP. Therefore, the increase in yields observed in this distal part, cannot be assigned to the use of the Fricke dosimeter in an inadequate domain of response. However, the observed decrease of dose rate can be the result of proton energy decrease and consequently the result of a lower LET value. This effect is identified and discussed by Garcia-Molina et al. in 2011 and De Vera et al. in 2018^45,46^. These authors provide some experimental data from other groups obtained essentially in ice water and vapour concerning the energy spread and stopping power around the proton Bragg peak and its maximum occurring for individual protons at 80 keV. In the 2011 article, they also show in Fig. 1, the curve presenting their calculation results by using the MELF-GOS model^45^. In Fig. 4, they show the energy decrease and energy spread increase as the protons are stopping, even in the distal part of BP^45^. Therefore, we propose to attribute the yield raise in the distal part of BP to the intermediate LET provided by the decrease of protons energy and their energy spread increase. This spread is accompanied by an increasing population of very low energy protons giving rise to lower LET-like effect, which are the origin of the increase of the Fricke yield in the distal part of BP. De Vera et al. evoked other phenomena of different nature of LET those could induce this effect at the nanoscale level. For the first time, it is observed by using a chemical system. Nevertheless, Fricke dosimeter yields for protons of few tens of keV have not been measured yet and we do not know what should be the influence of their LET value on the yield. Then we could consider this study provides now the first evaluation of the Fricke yield in the distal part of BP.

To shortly compare the above mentioned to the biological implications of the proton track-end straggling, it should be very similar to the recent observations of Horendeck et al.^30^ The authors examined microscopic DNA damage and revealed an extended cytotoxicity in CHO cells over a few millimetres after the BP^30^. They show that the BP and post-BP regions present high LET-like radiation tracks that can cause cellular lethality^30^. The consequences in proton therapy are potential side effects, which could lead to radiation-induced cancers. In the current study, we proposed another approach that clearly leads to a low LET–like effect, which probably means this effect has nothing to do with the LET.

Monte Carlo simulations have demonstrated their accuracy to reproduce radiolysis processes even in the energy range present in BP (i.e. very low energies)^19,47–49^. Since they provide similar ELF at the BP (Cf SI-4) they could serve in the depiction of the ferric ions yields as it was recently carried out under gamma rays^50^. This should be a good way to obtain the details in terms of radiolytic species and, consequently, to better understand the toxicity of ROS provided by elevated LET and extreme dose rates. Nevertheless, since the ferric ions yields reach a minimum value at the BP, one could also assume that the direct effect of proton is the main cytotoxic effect in radiobiology at the BP. We see then the interest to keep on with deeper studies at the microscopic scale to describe the fast processes in BP by using various swift ions starting with alpha rays that provide excellent results in vectorised therapy and by also investigating the enhancement effect of nanoparticles and the influence of radiosensitizers^17,51^.

## Conclusion

The super-Fricke chemical solution was used to investigate the radiolytic response of water under high-LET projectile stopping in 1 cm. An optical system has been set-up to follow the absorbance and the track-segment yield of a super-Fricke system along the ion propagation axis, including the BP. For proton beam, we show that the longitudinal straggling plays an important role by inducing a yield increase in the distal part of BP. We cannot assume this effect is brought by the combined effects of low dose rates and high LET protons because we demonstrated a dose rate effect is not credible for the super Fricke system for the considered dose rates all along the tracks in this current study. The key question is then: do we have high-LET protons in the distal part of the Bragg peak? Garcia-Molina et al. showed in this region that the energy of protons decreases accompanied by an increase of the energy spread, which corresponds to a lowering of the stopping power of water^45,46^. We propose to attribute that the rise of the Fricke yield in the millimetre region beyond the BP is due to intermediate LET caused by the proton beam straggling and more specifically to very low energy protons. Except for the collapse of the ferric ions at the BP, the early results obtained for carbon ions did not present similar behaviour in post-BP range because of a too sharp BP for our current spatial resolution. A microscopic resolution is to address in near future as it is presented as an improvement of the current optical setup. It should bring a better understanding of the chemistry in this ion track-end region. This study can therefore participate in answering questions about the validity of the constant RBE applied near the BP for proton treatment^32^. It should also contribute to the improvement of future modalities of hadrontherapy (proton-FLASH or PMBRT)^52^ in terms of spatial precision and efficiency. As the dose rates used in this study are similar to those encountered in FLASH modality^50^, it is interesting to evaluate a dose rate effect on specific ROS such as ^⋅^OH, by using another chemical system than Fricke dosimeter, for which the linearity domain is too large in terms of dose rate. Kusumoto et al. observed recently a dose rate effect on C3CA system that scavenges ^⋅^OH and due to the complex reaction mechanism needing O_2_ assistance, the authors also attributed the effect to O_2_ depletion^53^.

## Materials and methods

### Super-Fricke solution

The Fricke dosimeter is an aqueous solution allowing a convenient determination of doses delivered by ionizing particles in water when G-values are known^16,54^. This dosimeter action is based on the oxidation of Fe^2+^ into Fe^3+^ by ⋅OH, HO_2_⋅ and H_2_O_2_ produced by water radiolysis. Through the use of the oxidation of Fe^2+^ into Fe^3+^ the primary radiolytic yields have been very well documented since its first application for all types of ionizing radiation^16,39,55^.

In this study, we used the Super Fricke system to determine the yield of Fe^3+^ along ion tracks. Super Fricke solution is recommended when high intensity irradiations are used. As a matter of fact, high-LET radiation can act as a local high intensity irradiations because of the elevated density of ionization and excitation along the ion track^44,56^.

The chemical system of the Super-Fricke dosimeter is an oxygen saturated aqueous solution containing 10 mM of Fe(SO_4_)_2_(NH4)_2_,6H_2_O (Mohr’s salt) 0.4 M of H_2_SO_4_ and 1 mM of NaCl. The Mohr’s salt (Ammonium iron(II) sulfate hexahydrate, purity higher than 99.0%), which provides Fe(II), and the anhydrous sodium chloride (purity higher than 99.999%) were supplied by Sigma Aldrich, whereas sulphuric acid (purity higher than 96%) was supplied by Merck. All reagents were used without any further purification stage. The water used for all preparations was ultrapure water provided by a Millipore Milli-Q system (18.2 MΩ·cm, Total Organic Carbon of 3 ppb). Oxygen gas was an ultrapure gas provided by Gas Product. It was directly bubbled in the cuvettes containing the solution.

The oxidation process at stake of the Fricke solution is depicted via the mechanism presented below (reactions (1) to (5)). It is same for Super Fricke solution. Under oxygen saturation and acidic pH, e^–^_aq_ and ⋅H are first converted into HO_2_^⋅^ following reactions (1) and (2) before the Fe^2+^ oxidation takes place.

1 $${\mathrm{H}}_{3}{\mathrm{O}}^{+}+ {\mathrm{e}}_{\mathrm{aq}}^{-}\to {}^{\cdot }\mathrm{H\,\,\,\,\, k}=2.3\mathrm{ x }{10}^{10} {\mathrm{M}}^{-1}{\mathrm{s}}^{-1},$$

2 $${}^{\cdot }\mathrm{H}+ {\mathrm{O}}_{2}\to {\mathrm{HO}}_{2}^{\cdot }\,\,\,\,\,\mathrm{ k}=2.1\mathrm{ x }{10}^{10} {\mathrm{M}}^{-1}{\mathrm{s}}^{-1}.$$

Followed by the reactions of oxidation of Fe^2+^

3 $${\mathrm{Fe}}^{2+}+{}^{\cdot }\mathrm{OH}\to {\mathrm{Fe}}^{3+}+{\mathrm{OH}}^{-}\,\,\,\,\,\mathrm{ k}=4.3\mathrm{ x }{10}^{8} {\mathrm{M}}^{-1}{\mathrm{s}}^{-1},$$

4 $${\mathrm{Fe}}^{2+}+{\mathrm{H}}_{2}{\mathrm{O}}_{2}\to {\mathrm{Fe}}^{3+}+{}^{\cdot }\mathrm{OH}+{\mathrm{OH}}^{-}\,\,\,\,\,\mathrm{ k}=42 {\mathrm{M}}^{-1}{\mathrm{s}}^{-1},$$

5 $${\mathrm{Fe}}^{2+}+{\mathrm{HO}}_{2}^{\cdot }\to {\mathrm{Fe}}^{3+}+{\mathrm{H}}_{2}{\mathrm{O}}_{2}+{\mathrm{OH}}^{-}\,\,\,\,\,\mathrm{ k}=2.1\mathrm{ x }{10}^{6} {\mathrm{M}}^{-1}{\mathrm{s}}^{-1}.$$

Based on this mechanism, the observed yield of Fe^3+^ is a linear combination of the G-values of species formed during water radiolysis:

6 $$\mathrm{G}\left({\mathrm{Fe}}^{3+}\right)=\mathrm{G}\left({}^{\cdot }\mathrm{OH}\right)+2\mathrm{ G}\left({\mathrm{H}}_{2}{\mathrm{O}}_{2}\right)+3\left(\mathrm{G}\left({\mathrm{e}}_{\mathrm{aq}}^{-}\right)+\mathrm{G}\left({}^{\cdot }\mathrm{H}\right)+\mathrm{G}\left({\mathrm{HO}}_{2}^{\cdot }\right)\right).$$

G values are often given in molecules per 100 eV (1 molecule/100 eV corresponds to 1.036 × 10^–7^ mol·J^–1^).

Reactions (1) to (5) are completed within the microsecond regarding the reaction rates mentioned and the concentration used in Super Fricke solutions. In addition, the oxidized form Fe^3+^ exhibits an absorption spectrum in UV with a maximum at 304 nm, while the initial Fe^2+^ shows a low absorption background in this wavelength domain.

### Ion beams characteristics

Proton irradiations were performed on the R&D beam-line of the MEDICYC cyclotron (Centre Antoine Lacassagne, Nice, France). This beam-line generates proton beams with an energy of 64.0 MeV at the exit of the vacuum chamber. The energetic profile of the beam is Gaussian with an energy spread of 0.3 MeV. The beam current can have an intensity between < 1 pA and up to 10 uA. A proton beam was collimated to a diameter of 8 mm and its intensity was monitored in line by using an ionization chamber, which was calibrated with respect to the signal of a Faraday cup at the sample position. This installation benefits from well-described physical characteristics for its use in clinical protontherapy^57^. 25-MeV protons were generated by degrading the 64-MeV proton beam with a 23.57 mm degrader of PMMA located upstream of the irradiation sample and the 62-MeV were generated simply by using the air upstream the sample to degrade the 64-MeV protons. Irradiations were performed at several beam intensities (250 pA, 500 pA, 1 nA and 2 nA).

The carbon ion beams were delivered by the GANIL installation with an energy of 95 MeV/nucleus at the exit of the vacuum chamber. The beam-controller allowed a carbon beam diameter of 8 mm adjusted on an alumina target. The ions then passed through 14 mm of PMMA before the sample to slow down to approximatively 550 MeV. With this energy, the carbon ions stopped in the sample and the BP is located in the center of the 1-cm sample. A beam intensity of 100 pA was chosen to limit the dose rate effects due to the extreme high LET near the BP.

### Dose rate along the ion track

Each ion delivers a dose in the medium and in infinitesimal volumes on its axis of propagation. In order to be able to determine the radiolytic yield of the Fricke system, this dose or dose rate must be evaluated as accurately as possible. This was achieved thanks to calculation codes. For the general purpose particle–matter simulation codes and libraries, FLUKA^58^ and GEANT4^59^ are two of them among several ones which were discussed in a recent review^48^. They are used for dose determination in proton therapy. These codes are based on the Monte Carlo method and on the use of the ionization/excitation cross sections as a function of the incident energy. SRIM, another code, is based on ion-range tables and on the Bethe-Bloch theory. Unlike other codes, the geometry of the crossed materials is described only by their thickness. Nevertheless these different methods offer several approaches that are mature enough to be compared and used in dosimetry^8,60^. They also take into account the energy-loss straggling (angular and longitudinal) which generally induces larger BP.

The dose rate estimation, dD/dt, was first calculated from the energy loss function (ELF) given by TRIM as a function of distance on the propagation axis of the particle. We have reasonably neglected the angular straggling after the collimating optics, with respect to the volume impact and dose effect. We also used GEANT4^59^ and FLUKA^58^ for comparison of dose rate values and position and shapes of the BP. The calculation was made assuming a constant volume, i.e. a cylinder of solution with a constant section corresponding to the collimation of 8 mm. We finally used the dose rates given by GEANT4 for the G-values determination. We report, in the supplementary information section (Supplementary Information), the ELF of protons and carbons in Super Fricke solution (density of solution of 1.024 g/cm^3^) along the last mm of the ion propagation axis. We considered all thicknesses of all material crossed by the beam from the exit window of the accelerator to the end of the 10-mm sample.

### In line optical setup

An optical setup was designed to measure the optical absorption variations A(t, x) of the radiation-induced Fe^3+^ at a position x in the propagation axis of the ion beam (Fig. 4). We used the 30 mm Cage system from Thorlabs to build our set-up. A bundle of 20 optical fibres (IDIL) with a 250 µm diameter was implemented to measure the transmitted light on 20 different portions of the track at a given time. This allowed us to capture the BP and the events occurring just before it. The light source used for transmission/absorption spectroscopy was a white laser-driven light source, LDLS™ provided by Energetiq. The light intensity was conducted to the entrance of the optical setup with a 25-m long optical fibre of 600-µm diameter connected to an optical fibre adapter with a converging lens. An optical filter was placed at the exit of the adapter to select the wavelength of interest for Fe^3+^ absorbance: 304 nm (Interference filter provided by MKS, model 10BPF10-300). A spatial filter with a 5 mm diameter aperture was positioned and a system of converging lens including a cylindrical lens (focus length of 3 cm) focused the light on the ionizing particles pathway in the middle of the sample. The sample was placed in a cuvette holder that was able to receive 10 × 10 × 45 mm Plexiglas cuvette. Apertures on the sides, front and back of the sample holder let the light and the irradiating beam pass through the sample.

The transmitted light was collected by another system of converging lens producing a magnification of 8/9 on the image plane where the linear bundle of 20 optical fibres is settled. With a magnification of 8/9, the space observed by a 250 µm diameter fibre was a 281.25 µm wide interval. The 25-m long fibre bundle transmitted the collected light of every fibre to the entrance slit of a monochromator (Omni-λ 200i, Zolix). The linear fibre bundle was perfectly aligned with the slit to obtain the best monochromator transmission. A sCMOS camera (Orca-Flash4.0, Hamamatsu) was connected and aligned at the exit focal plane of the monochromator. The monochromator dispersed horizontally the light to the camera focal plane centered at 304 nm. Thus, the vertical axis of the camera’s image corresponds to the number of the bundle’s optical fibers (from 1 to 20), which refers to their position on the axis of propagation of the ion beam. The camera recorded sequences of images with an integration time of 100 ms and a duration of 1 min. The sequences were recorded with the maximum resolution allowed by the camera binning (2048 × 2048 for a binning 1 and 1024 × 1024 for a binning 2). The sequences were saved with a binning of 1 and a binning of 2 for proton and carbon irradiations respectively. A robotic arm (Ned, NIRYO) changed the sample between each irradiation sequence, which greatly increased the productivity during the experiments. The sample was not stirred during irradiation.

### Image analysis

The image sequences were analysed with ImageJ program. It is a multithreaded program, which greatly accelerates the processing of image sequences, which in the present case can easily reach a size of 1 Go corresponding to 600 images.

Details of data treatment are reported in SI-2 and show how a rough recorded image sequence is processed leading to the variation in time of absorption *A* (304 nm) for each position in the propagation axis of ions.

### Yield determination

By measuring the variation of absorption of Fe^3+^ at 304 nm over time during an irradiation, we were able to determine G(Fe^3+^). The general formula to calculate the radiolytic yield of a species X is given by the following equation^7^.

7 $$\begin{array}{c}G\left(\mathrm{X}\right)=\frac{1}{\uprho }\cdot \underset{\mathrm{D}\to 0}{\mathrm{lim}}\frac{\mathrm{d}C\left(\mathrm{X}\right)}{\mathrm{d}D} .\end{array}$$

With G(X) in mol/J, *C*(X) the concentration of X in mol/dm^3^, *D* the dose in Gy and ρ the density of the irradiated matter in kg/dm^3^. In the case where the measured parameter is the absorbance and where the delivered dose rate is constant during time (starting at t = 0 s), Eq. (7) can be reformulated as follow using the Beer-Lambert law:

8 $$\begin{array}{c}G\left(\mathrm{X}\right)=\frac{1}{\uprho \cdot \upvarepsilon \cdot l\cdot \frac{\mathrm{d}D}{\mathrm{d}t}}\cdot {\left(\frac{\mathrm{d}A}{\mathrm{d}t}\right)}_{t=0} ,\end{array}$$

where* ε* is the extinction coefficient of Fe^3+^ at 304 nm (2212 M^–1^.cm^–1^ at 25 °C), ρ is the density of the Fricke solution (1.024 g/cm^3^) and *l* is the length of the optical path in cm. In this case, *l* = 0.8 cm because Fe^2+^ oxidation occurs in the ionizing beam of 0.8 cm of diameter. The variation of absorbance d*A*/d*t* at *t* = 0 is evaluated from the recorded *A*(*t*). The timestamp *t* = 0 is defined as the moment when the irradiation starts.

It is crucial to determine the yields in the first seconds of the irradiation (i.e. less than few tens of image acquisitions) to respect the definition given by Eq. (8) and to be allowed to neglect diffusion phenomena. In order to do so, we have drawn a linear regression on the first points following the start of the irradiation. The number of points used to make this linear regression is also a compromise between having the lowest number of point to be the closest to ${\left(\frac{\mathrm{d}A}{\mathrm{d}t}\right)}_{t=0}$ and having the highest number of points to get the correlation factor r^2^ the closest to 1. In the case of the proton beam we used the 10 first points (i.e. within 1 s) and in the case of the carbon beam we used the 15 first points (i.e. within 1.5 s). The slope of the linear regression gave ${\left(\frac{\mathrm{d}A}{\mathrm{d}t}\right)}_{t=0}$. Then G(Fe^3+^) was deduced from it and from the calculated dose rate d*D*/d*t* according to Eq. (8).

The continuous dose rate d*D*/d*t* (in Gy/s) is evaluated through calculations using the ELF of the ionizing particle and the beam intensity *I* (in A) for every position along the propagation axis x:

9 $$\begin{array}{c}{\left(\frac{\mathrm{d}D}{\mathrm{d}t}\right)}_{x} =\frac{ELF (x)\times I}{1.6\cdot {10}^{-19}}.\end{array}$$

Equations (8) and (9) lead to the expression of the differential Fricke yield at the position x on the propagation axis:

10 $$\begin{array}{c}{\left\{\mathrm{G}\left({Fe}^{3+}\right)\right\}}_{x}=\frac{1.6\cdot {10}^{-19}}{\uprho \cdot \upvarepsilon \cdot l\cdot ELF(x)\cdot I}\cdot {\left(\frac{\mathrm{d}A}{\mathrm{d}t}\right)}_{t=0} .\end{array}$$

### Supplementary Information

Supplementary Information.

## Data Availability

The datasets generated during and/or analysed during the current study are available from the corresponding author on reasonable request. All data generated or analysed during this study are included in this published article (and its Supplementary Information files).
