# Supplementary material for: Intermediate LET-like effect in distal part of proton Bragg peak revealed by track-ends imaging during super-Fricke radiolysis
Source: Sci Rep. 2023 Sep 19;13:15460. doi: 10.1038/s41598-023-42639-4 (PMC10509149; doi:10.1038/s41598-023-42639-4)
Supplement: Supplementary file 1 — Supplementary Information. [file 41598_2023_42639_MOESM1_ESM.docx]

**Supplementary Information**

**Intermediate LET-like Effect in Distal Part of Proton Bragg Peak Revealed by Track-Ends Imaging during Super-Fricke Radiolysis.**

**Authors:**

Audouin J.^1^, Hofverberg J.P.^2^, Ngono-Ravache Y.^3^, Desorgher L.^4^, and Baldacchino G. ^1#^

**SI-1 Determination of the Energy Loss Function (ELF) for proton and carbon beams**

The ELF was determined by simulation using GEANT4 (Geant4.11.0) and for comparison using FLUKA (FLUKA CERN 4.2.2) and TRIM (Version SRIM2008). We considered that a proton beam of 64 MeV or a carbon beam of 1140 MeV crosses all materials. The details are given in SI-1 Figure 1. The SI-1 Table 1 gives the parameters of each material crossed by the beam. In this Figure, we consider the beam starts in the vacuum tube where the particles have their initial energy. The proton beams were generated with an initial energy of 64 MeV and the carbon beams with an initial energy of 1.14 GeV. Using the dedicated absorbers, we could localise the Bragg Peak (BP) roughly in the middle of the 1-cm thick Fricke solution.

The simulations made with Geant4 and Fluka for the proton beam used a pencil-beam with a Gaussian energy distribution. The mean initial energy was 64.0 MeV with a variance of 0.3 MeV^1^. The beam interacted with a sample made of pure water with a density of 1.024. The spatial resolution was 50 µm. The ELF was also given as a mean value on a disc with a diameter of 8 mm.

The simulations for the carbon beam also used a pencil beam but with no Gaussian energy distribution. The spatial resolution was 50 µm.

GEANT4 and FLUKA simulations are presented in SI-1 Figure 2.

TRIM simulations used the stopping power included in the SRIM-2008 program version. The damages were calculated with an ion distribution and a quick calculation of damage. The angle of incidence was 0° for every simulation. We used the default data provided by TRIM for the ionizing beams. The initial energy of the carbon beam was set to 1.14 GeV. The final ELF curves is obtained with TRIM by considering the energy loss in layers unlike GEANT4 and FLUKA which provide the dose in a 3D view by accounting collimation and cylindrical shape of the beam. The ELF was calculated as a mean value of 10 000 shots of carbon. TRIM was used mainly to evaluate the range of protons and carbon ions in the experimental setup but it cannot be used for accurate yield evaluations because it does not account for the geometry of the beam and its spectral range.


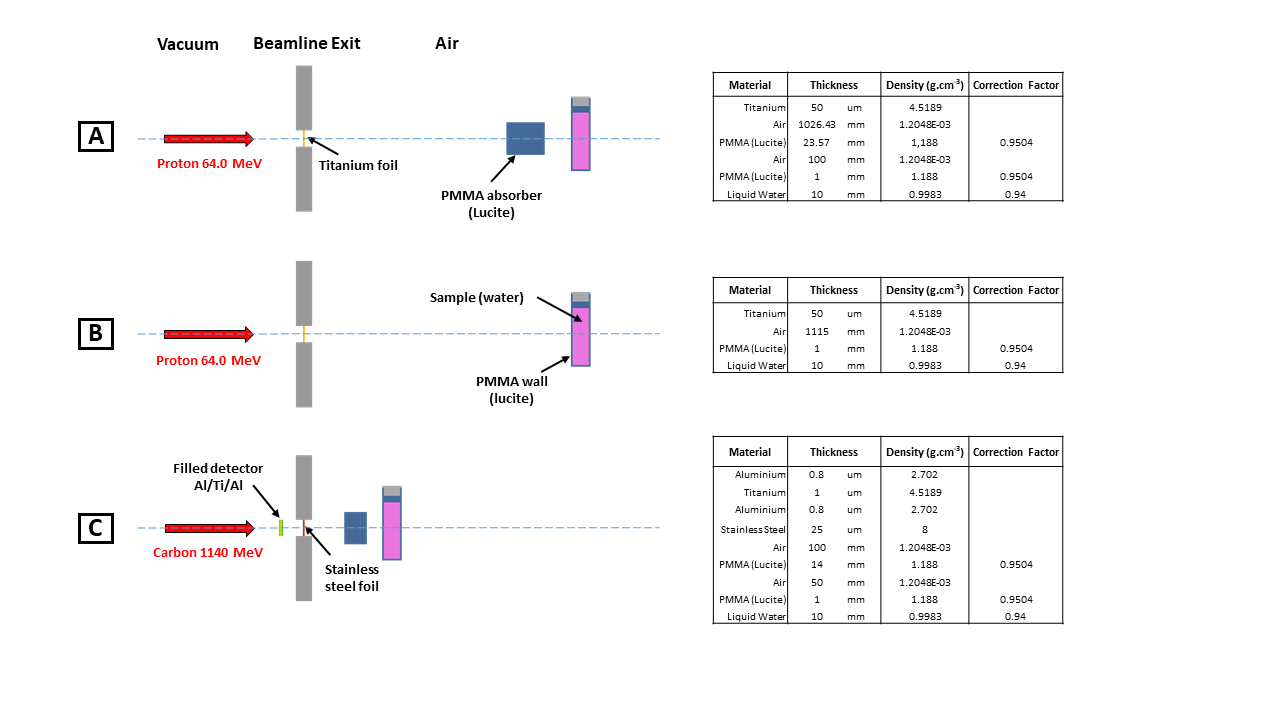


**SI-1 Figure 1:** Schematic of the trajectory of ionizing beams. [A] Trajectory of 25-MeV proton beam; [B] Trajectory of 62-MeV proton beam and [C] Trajectory of 550-MeV C^6+^ beam. PMMA absorber (Lucite) in the material list of TRIM program, were used in [A] and [C] to decrease the energy of the particles and to obtain 25-MeV protons and 550-MeV carbons at the entrance of the sample cuvette.

**SI-1 Table 1**: *Characteristics of thickness and density of the materials crossed by the beams*.

| **A (25-MeV proton)** | | | |
| --- | --- | --- | --- |
| **Material** | **Thickness** | | **Density (g.cm-3)** |
| Titanium | 50 | µm | 4.5189 |
| Air | 1026.43 | mm | 1.2048E-03 |
| PMMA (Lucite) | 23.57 | mm | 1.188 |
| Air | 100 | mm | 1.2048E-03 |
| PMMA (Lucite) | 1 | mm | 1.188 |
| Liquid Water | 10 | mm | 1.024 |
|  |  |  |  |
|  |  |  |  |
| **B (62-MeV proton)** | | | |
| **Material** | **Thickness** | | **Density (g.cm-3)** |
| Titanium | 50 | µm | 4.5189 |
| Air | 1115 | mm | 1.2048E-03 |
| PMMA (Lucite) | 1 | mm | 1.188 |
| Liquid Water | 10 | mm | 1.024 |
|  |  |  |  |
|  |  |  |  |
| **C (550-MeV C^6+^)** | | | |
| **Material** | **Thickness** | | **Density (g.cm-3)** |
| Aluminium | 0.8 | µm | 2.702 |
| Titanium | 1 | µm | 4.5189 |
| Aluminium | 0.8 | µm | 2.702 |
| Stainless Steel | 25 | µm | 8 |
| Air | 100 | mm | 1.2048E-03 |
| PMMA (Lucite) | 14 | mm | 1.188 |
| Air | 50 | mm | 1.2048E-03 |
| PMMA (Lucite) | 1 | mm | 1.188 |
| Liquid Water | 10 | mm | 1.024 |







**SI-1 Figure 2:** *ELF curves calculated with FLUKA, Geant4 and TRIM for proton and carbon beam interacting with a Fricke solution of density 1.024 g/cm^3^*. [A] ELFs calculated with a 62-MeV proton beam, [B] ELFs calculated with a 550-MeV carbon beam. Fluka and Geant4 ELFs are normalized for each ion exiting the collimator.

**SI-2 Data treatment of image sequences**

The image sequences were imported in ImageJ as a stack of matrix. In order to convert the temporal image sequences into spectral image sequences, where each image corresponded to a given wavelength, the data were reorganized using the function “Reslice” from the module “Stacks”. The following parameters were used: 1- the image-sequence treatments start from the left of the pictures, 2- an output spacing of 1 inch, an output rotation of 90 degrees, 3- no interpolation and no vertical flip were applied. Since the original images displayed the light intensity spectrum along the track of the ionizing particles, the newly obtained images displayed the light intensity during time (going from the left to the right of each picture) along the track, at a given wavelength. Thus, by selecting and processing a specific image of the spectral sequences, we were able to directly obtain the light intensity as a function of time for a specific wavelength (here 304 nm) for each optical fibre along the track.

Then, we defined for each optical fibre and the background a Region Of Interest (ROI) with a size of 601x16 for pictures with a binning 2 and of 601x32 for pictures with a binning 1. The ROI for the background was positioned at the TOP of the image where there was no light transmitted by the optical fibres (only the background light). With a multiplot, the intensities of light during time were extracted from the 21 ROI in a tabular ASCII file. SI-2 Figure 1 summarizes all these process. Then, we can observe a diminution of the light intensity on the right part of the picture due to the increase of absorption that results from the irradiation.


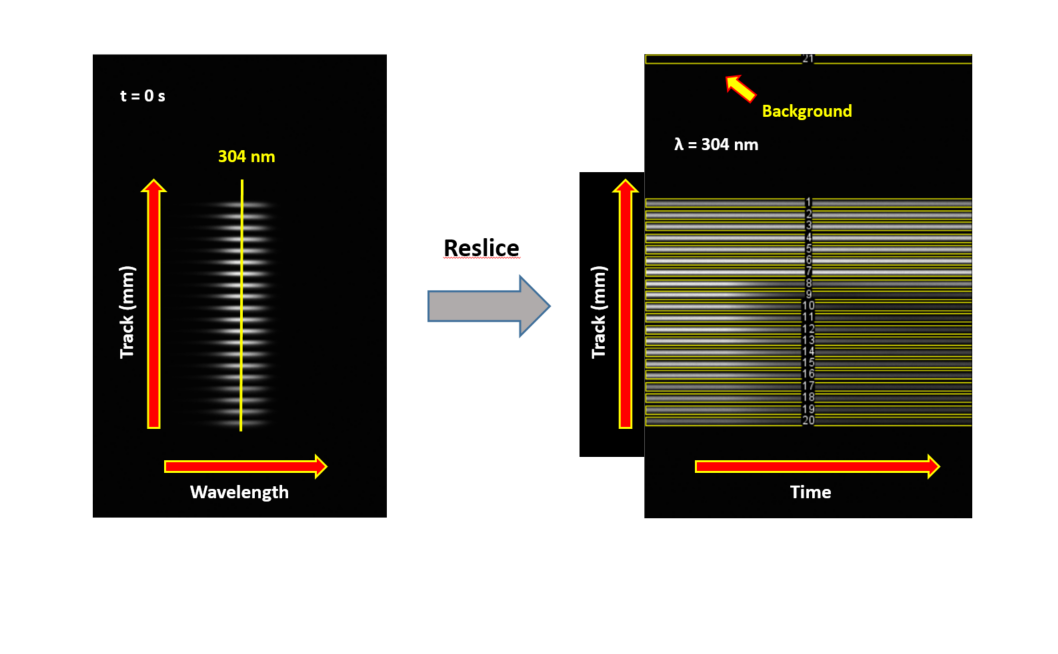


**SI-2 Figure 1**: ImageJ conversion using “Reslice” function, of a temporal sequence of pictures into a spectral sequence. Left: picture taken at t = 0 s, it displays the transmitted-light spectrum from the Fricke solution and the bandpass filter at 300 nm. Right: picture obtained after the reslice of the temporal sequence, it displays the transmitted light intensity in time along the propagation axis, at 304 nm.

The table obtained with ImageJ was then processed using a data analysing and graphing software. The mean temporal background was subtracted to the raw light intensities during time to get net intensities during time. In addition, the absorption during time was calculated with the following formula:

$$A\left( t \right)=-log(\frac{I\left( t \right)}{I_{0}})$$

Where A(t) is the absorption during time. I(t) is the intensity of light during time and I_0_ is the initial intensity of light before irradiation. I_o_ was calculated as the mean value of the 20 first values of light intensity for each sequence, which corresponds to the first 2 seconds of each sequence where the irradiation of the sample has not started yet. Following the calculation, A(t) was plotted.

**SI-3 Calculation of the mean ELF for a fibre**





**SI-3 Figure 1**: The red dots represent the normalized variation of absorption during the 2 first seconds of an irradiation. Each dot is the mean value of two measures performed during two different irradiations with the same beam parameters, which were here a 25-MeV proton beam with an intensity of 0.5 nA. The black dots are the ELF curve calculated by Geant4 for a 25 MeV proton beam. The blue vertical line represent the position of the external side of the optical fibres. The length comprised between each blue vertical line represents the portion of the track monitored by one optical fibre. The fibre 20 was set at the earliest time in the track while fibre 1 is the farthest fibre in the track.

When we determine the yield of Fe^3+^ on a fibre, we determine a segment track yield on a portion of the track with a length of 0.28125 mm. In order to calculate this segment track yield, we need to evaluate the mean ELF value on the fibre from the ELF curve calculated with Geant4 (respectively with Fluka). We calculate the mean value of ELF on each segment through an integration of the ELF curve on the corresponding segment divided by the diameter of the fibre. The integrations are performed using the trapezoidal approximation.

**SI-4 Comparison of the yields obtained for proton beams with Geant4 and Fluka**

The track-segment yields presented in SI-4 Figure 1 and SI-4 Figure 2 were computed similarly to the one presented in the results part of this article. SI-1 Figure 2-A shows that the ELF given by Geant4 and Fluka overlap in the sample for the high energies. Thus, SI-4 Figure 1 displays no significant difference between the yields calculated with Geant4 and Fluka. However, SI-1 Figure 2-A also shows that the ELF of proton beam with a 25-MeV energy at the entrance of the sample presents some differences between Geant4 and Fluka. The BP calculated by Geant4 appears a fraction of millimetre before in the track and with a lower maximum compared to Fluka BP respectively position and maximum. This difference can have significant repercussions on the values of the yields. On SI-4 Figure 2, B.2 and B.3 display yields that decrease in the Bragg peak. No significant raise after the ELF maximum is observed. This discrepancy is due to the strong variation of the ELF in the Bragg peak. Consequently, a change of its calculated position from one software to another can strongly affect the global behaviour of the yield curve as it can be seen on B.2 and B.3. Nevertheless, B.2 shows this sensitivity depends on the series of experimental acquisition since black data reveals a raise after BP and the red one none. The positioning of the cuvette and the thickness of Lucite thickness of the cuvette can change from an acquisition to another. So statistically speaking, the global trend of these experiments reveal a raise of yield after the BP.





**SI-4 Figure 1**: *Track-segment yields of Fe^3+^ for 62-MeV proton beams*. Beam intensities: () 0.25; () 0.5; () 1 and () 2 nA. [A] and [B] respectively represents the yields calculated with the ELF given by Geant4 and Fluka. Error bars are displayed for each point.





**SI-4 Figure 1**: *Track-segment yields of Fe^3+^ for 25-MeV proton beams and for 4 intensities of beam*. Two experimental series ( and ) treated with the same parameters. [A.1] (respectively [A.2], [A.3] and [A.4]) represents the track-segment yields obtained for proton beam with an intensity of 0.25 nA (respectively 0.5 nA; 1 nA and 2 nA) and using Geant4 ELF. [B.1] (respectively [B.2], [B.3] and [B.4]) was obtained for proton beam with an intensity of 0.25 nA (respectively 0.5 nA; 1 nA and 2 nA) using Fluka ELF. The normalized ELF are also displayed for Geant4 series (A.1 to A.4) and for Fluka series (B.1 to B.4).

**SI-5 Estimation of the error bars on the position and on the value of the yields**

Estimations of the error on the position of the yields have been made for the proton beam. We identified the main causes of this error. They are the initial mean energy of the beam and the thickness of the plastic wall of the cuvette. The initial energy variation of the proton beam is known to be less than 0.1 MeV from one shot to another. Therefore, we chose to inflate this variation with this value. We measured the thickness of 10 different cuvettes to evaluate the variation of thickness of their plastic wall. We found a variation of 0.1 mm. With TRIM, we estimated the impact of these variations on the position of the Bragg peak. We calculated the ELF in the 2 worst case scenario. The first one is a beam of 63.9 MeV with a plastic thickness of 1.1 mm and the second one is a beam of 64.1 MeV with a plastic thickness of 0.9 mm. With this method, we found an error 0.14 mm for the position of the yield.

The evaluation of the errors on the yield’s values could not be performed with the use of statistics due to the small amount of yields obtained for each set of parameters. Instead, we chose to evaluate the error of each parameter susceptible to significantly impact the yield. We found out that the beam intensity and the variation of absorption during time have by far the most impact on the error. The other parameters in the yield calculation have a negligible influence on it. The relative error of the beam intensity is known to be 5%^1^. The relative error on the absorption variation during time was assessed using the error calculated by OriginPro for the linear regression. The resulting errors on the yield have been calculated with the following formula.

$$\Delta G=\Delta I+\Delta\frac{\mathrm{dA}}{\mathrm{dt}}$$

Where ΔG is the relative error on the yield value, ΔI is the relative error on the beam intensity and ΔdA/dt is the relative error on the absorption’s variation.

References

1. Hofverberg, P. *et al.* A 60 MeV proton beam-line dedicated to research and development programs. *Appl. Radiat. Isot.* **184**, 110190 (2022).
